# Disruption of Man-6-P-Dependent Sorting to Lysosomes Confers IGF1R-Mediated Apoptosis Resistance

**DOI:** 10.3390/ijms26083586

**Published:** 2025-04-10

**Authors:** Asena Aynaci, Maxence Toussaint, Florentine Gilis, Martine Albert, Jean-François Gaussin, Michel Jadot, Marielle Boonen

**Affiliations:** 1 Laboratory of Intracellular Trafficking Biology, URPhyM, NARILIS, UNamur, 61 rue de Bruxelles, 5000 Namur, Belgium; asena.aynaci@unamur.be (A.A.); maxence.toussaint@unamur.be (M.T.); florentine.gilis@unamur.be (F.G.); martine.albert@unamur.be (M.A.); jean-francois.gaussin@unamur.be (J.-F.G.); 2 Laboratory of Physiological Chemistry, URPhyM, NARILIS, UNamur, 61 rue de Bruxelles, 5000 Namur, Belgium; michel.jadot@unamur.be

**Keywords:** lysosomes, *GNPTAB*, mucolipidosis II, apoptosis, IGF1R, mannose-6-phosphate, M6PR

## Abstract

Mutations in *GNPTAB* underlie mucolipidosis II and mucolipidosis III α/β, which are inherited lysosomal storage disorders caused by a defective UDP-N-acetylglucosamine:lysosomal-enzyme N-acetylglucosamine phosphotransferase. As a result, newly synthesized acid hydrolases fail to acquire Mannose-6-Phosphate (Man-6-P) sorting signals, or do so to a lesser extent, and exhibit an impaired trafficking to lysosomes. Interestingly, we found that *GNPTAB* knockout HeLa cells are resistant to several cytotoxic agents: doxorubicin, chloroquine, staurosporine and paclitaxel. While we detected an increased trapping of weak bases in the expanded lysosomal population of these cells, which could reduce the effect of doxorubicin and chloroquine; the decreased cell response to staurosporine and paclitaxel suggested the involvement of alternative resistance mechanisms. Indeed, further investigation revealed that the hyperactivation of the Insulin-like Growth Factor 1 Receptor (IGF1R) pathway is a central player in the apoptosis resistance exhibited by Man-6-P sorting deficient cells.

## 1. Introduction

Newly synthesized lysosomal acid hydrolases acquire mannose-6-phosphate (Man-6-P) residues on their N-glycans during transport through the Golgi apparatus. These Man-6-P residues are recognized by Man-6-P receptors (M6PR) located in the *trans*-Golgi Network (TGN). This association is a critical step in the subsequent sorting of the acid hydrolase to the endosomes, from where it can be sorted to lysosomes [1,2,3,4]. Man-6-P synthesis is a two-step process. The first step takes place in the *cis*-Golgi network: N-acetylglucosamine-1-phosphate (GlcNAc-1-P) is transferred at position 6 of a terminal mannose residue by a GlcNAc-1-phosphotransferase (uridine 5’-diphosphate-N-acetylglucosamine: lysosomal enzyme N-acetylglucosamine-1-phosphotransferase) [5,6]. The second step takes place in the TGN and is mediated by the uncovering enzyme (GlcNAc-1-phosphodiester α-N-acetyl-glucosaminidase). This enzyme excises the GlcNAc, leaving the phosphate group on the mannose, thereby generating the Man-6-P residue [7].

GlcNAc-1-phosphotransferase is a membrane-associated α_2_β_2_γ_2_ hexamer. The α and β subunits comprise the catalytic site and are encoded by the *GNPTAB* gene, whereas the γ subunit has a role in the presentation of hydrolase substrates to the catalytic site and is encoded by *GNPTG* [8]. Mutations in *GNPTAB* that cause a complete loss of GlcNAc-1-phosphotransferase activity underlie the lysosomal storage disorder mucolipidosis II (also called I-cell disease) [9,10]. Mucolipidosis III α/β and III γ are milder forms of the disease due to the residual GlcNAc-1-phosphotransferase activity [10,11]. These mucolipidoses II and III disorders are characterized by notable growth and developmental delays, skeletal abnormalities, facial dysmorphism and cardiomegaly. At the molecular level, acid hydrolases no longer acquire the Man-6-P signals required for the sorting to lysosomes (or do so to a lesser extent) and are therefore primarily secreted outside the cells, leading to the abnormal storage of non-degraded macromolecules in enlarged lysosomes and autolysosomes [12,13].

Interestingly, it has been reported that fibroblasts isolated from mucolipidosis II patients are resistant to drug- and stress-induced apoptosis [14,15]. This was demonstrated in several conditions, including treatment with MSDH (Methylmalonate Semialdehyde DeHydrogenase), naphthazarin, staurosporine, sphingosine and TNF (Tumor Necrosis Factor). Since the inhibition of cysteine and aspartic cathepsins (proteases located in lysosomes) conferred apoptosis resistance to control fibroblasts treated with several of these molecules, it was proposed that mucolipidosis II fibroblasts may be more resistant due to a decreased level of acid hydrolases, including cathepsins, in their lysosomes [14]. Indeed, the release of cathepsins from the lysosomal lumen to the cytosol is known to induce apoptosis [16,17]. For example, the cysteine protease cathepsin B can degrade a subset of anti-apoptotic factors (including members of the Bcl-2 family such as Bid), leading to the release of cytochrome c from the mitochondria and subsequent apoptosome assembly. According to Hanewinkel et al., mucolipidosis II fibroblasts secrete 98% of the newly synthesized cathepsin B precursors, which means that the amount of cathepsin B releasable from lysosomes under pro-apoptotic stress in these cells must be very low [18]. Cathepsin D, an aspartic protease, can also participate in apoptotic pathways [19]. However, it was documented that cathepsin D levels remain near normal in mucolipidosis II fibroblasts, most likely due to the Man-6-P-independent sorting of this hydrolase to lysosomes [15,20,21].

*GNPTAB* knockout (KO) HeLa cells have been used as a model of mucolipidosis II. They exhibit hypersecretion of lysosomal enzymes due to the deficient Man-6-P synthesis and contain enlarged lysosomes [22]. Here, we report that these cells resist cell death induced by doxorubicin-, staurosporine, chloroquine or paclitaxel. Our results are concordant with doxorubicin and chloroquine, which are weak bases, being efficiently trapped in the acidic environment of lysosomes, which are more numerous and enlarged in *GNPTAB* KO cells than in parental HeLa cells. However, we also discovered that the ratio of the phosphorylated/activated form of the Insulin-like Growth Factor Receptor 1 (IGF1R) to total IGF1R is higher in the cells with defects in Man-6-P sorting, making them resistant to the four studied drugs.

## 2. Results

### 2.1. GNPTAB Knockout HeLa Cells Exhibit Resistance Against Several Cytotoxic Molecules

**Figure 1:**
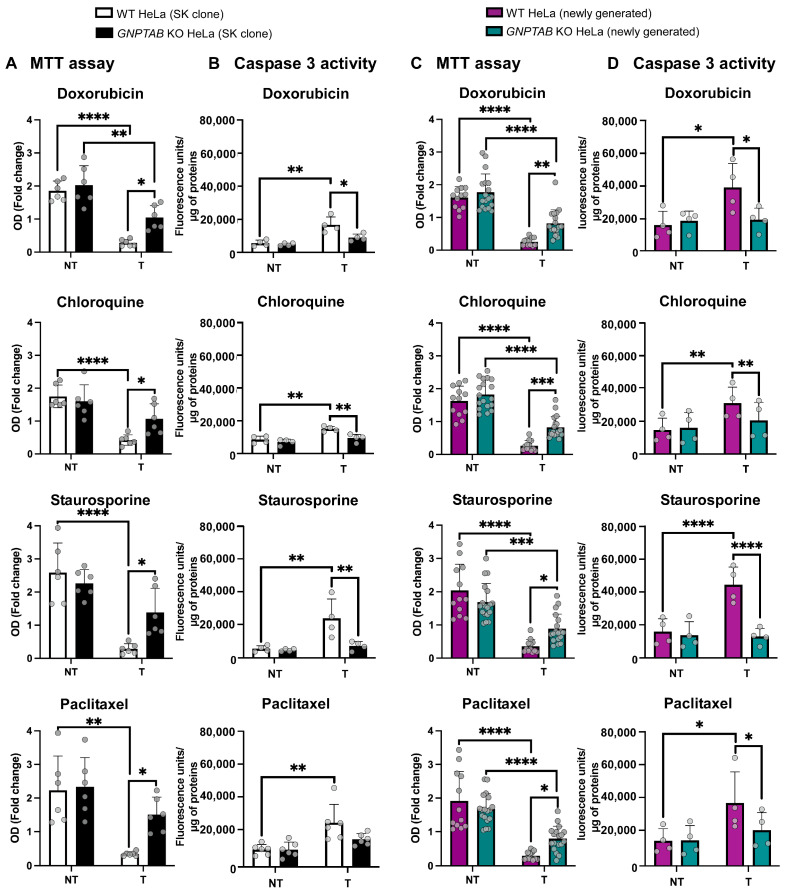
**Analysis of *GNPTAB* KO HeLa cells resistance to cytotoxic drugs.** (**A**,**C**) Measurement of metabolic activity of WT and *GNPTAB* KO cells ((**A**): SK clones and (**C**): newly generated clones) using MTT assay, 48 h after treatment with 2.5 μM doxorubicin, 25 μM chloroquine, 50 nM staurosporine or 50 nM paclitaxel. Means ± SD of OD_48h_ relative to OD_0h_ are shown on graphs. OD = Optical Density. n = 6 independent experiments for each panel (indicated with small circle dots). For panel C, 2 control and 3 KO clones were assessed separately in experiments prior to pooling of results. (**B**,**D**) Caspase 3/7 activity (fluorescence units/μg of total proteins) was measured in WT and *GNPTAB* KO cells ((**B**): SK clones and (**D**): newly generated clones), as described in Section Materials and Methods to assess apoptosis induction at early time points after treatment with doxorubicin, chloroquine, staurosporine or paclitaxel. Means ± SD. n ≥ 4 independent experiments (indicated with small circle dots). NT = Non-Treated and T = Treated * *p* < 0.05; ** *p* < 0.01; *** *p* < 0.001; **** *p* < 0.0001; and One-way ANOVA.

Control (Wild-Type, WT) and *GNPTAB* KO HeLa cells, generously provided by the group of S. Kornfeld (Washington University in St. Louis, MI, USA) and hereafter referred to as “SK clones”, were treated for 3 days with four drugs independently: doxorubicin, chloroquine, staurosporine or paclitaxel. The remaining metabolically active cells were analyzed daily by MTT assays, and the results obtained after 48 h of treatment are shown in Figure 1A. Without treatment no change in the metabolic activity (Figure 1A) or proliferation rate (Supplementary Figure S1, see non-treated conditions in panel A) was detected between WT and *GNPTAB* KO cells, which is consistent with the findings of the Kornfeld’s group [22]. However, in all treated conditions, the *GNPTAB* KO HeLa cells exhibited a higher residual metabolic activity compared to WT cells, indicating a resistance to the four drugs (Figure 1A, *p* < 0.05 for all drugs). This resistance was evident as early as 24 h after treatment (see complete survival curves from day 1 to 3 in Supplementary Figure S1).

Since these drugs are known to induce apoptosis, we next measured the specific activity of caspases 3/7, proteases that are activated in both intrinsic and extrinsic apoptosis pathways, and found that it increased to a lesser extent in *GNPTAB* KO HeLa cells compared to control cells after the indicated treatments (Figure 1B, *p* < 0.01, except for paclitaxel, for which the difference did not reach statistical significance).

To ensure that this resistance to the four drugs was not the result of a clonal effect, we generated additional *GNPTAB* KO HeLa clones (and corresponding controls) in our laboratory using the CRISPR-Cas9 editing strategy described in the Materials and Methods Section and illustrated in Supplementary Figure S2. This figure includes a description of the resulting mutational profile and analyses, demonstrating the absence of Man-6-P synthesis in the newly generated KO cells, causing acid hydrolases hypersecretion and lysosomal enlargement. Two control clones and three *GNPTAB* KO clones were selected to test the effect of the drugs. Figure 1C (MTT assays) and 1D (caspase assays) show that, similarly to the SK clones, the newly generated *GNPTAB* KO clones exhibit a resistance to doxorubicin, chloroquine, staurosporine and paclitaxel and decreased caspases 3/7 activity after treatment relative to control cells. Of note, the graphs show the pooled data obtained for the two control and three *GNPTAB* KO clones. Complete survival curves showing the drug response from day 1 to 3 are provided in panel B of Supplementary Figure S1.

### 2.2. Investigation of Putative Resistance Mechanism(s)

**Figure 2:**
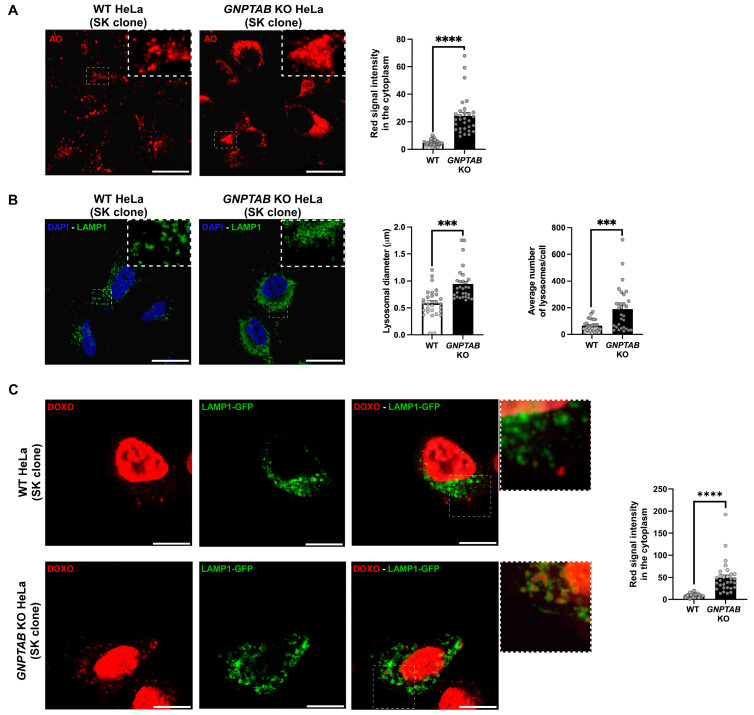
**Trapping of weak bases in *GNPTAB* KO and control HeLa cells.** (**A**) Labeling of intracellular acidic compartments of control and *GNPTAB* KO cells (SK clones) for 15 min with 1.5 μM acridine orange (AO). Scale bars, 25 μm. Graph shows mean red signal intensity in cytoplasm (arbitrary units) ± SEM. n = 3 independent experiments with 10 cells analyzed per genotype (shown as circled dots). (**B**) Immunofluorescence detection of late endosomal/lysosomal marker LAMP1 (green). Nuclei were stained with DAPI (blue). Scale bars, 25 μm. Graphs show mean lysosomal diameter and average number of lysosomes per cell ± SEM. n = 3 independent experiments with 10 cells analyzed in each experiment and for each cell type. (**C**) Detection of doxorubicin (DOXO, red) in cells transfected with LAMP1-GFP (green). Cells were observed 48 h post-transfection. They were treated for last 16 h with 2.5 μM DOXO. Scale bars, 10 μm. n = 3 independent experiments with 10 cells analyzed per condition. Graph shows mean red signal intensity in cytoplasm (arbitrary units) ± SEM. *** *p* < 0.001; **** *p* < 0.0001. Unpaired *t*-test. N.B. Dotted lines indicate zoomed areas.

Since doxorubicin and chloroquine are weak bases, we wondered whether these drugs exhibited a decreased activity in *GNPTAB* KO HeLa cells due to their confinement in lysosomes. Indeed, it has long been known that weak bases can diffuse into the lysosomes where the protonation occurs due to the low lysosomal pH, thus preventing them from leaving this organelle [23]. Acridine orange is a weak base probe that fluoresces red when protonated. Interestingly, *GNPTAB* KO HeLa cells accumulated approximately ~5 times more of this dye in their acidic compartments than control HeLa cells (Figure 2A). This correlates with the elevated number and size of the lysosomes in these KO cells compared to WT cells, as shown by the immunofluorescence detection of the late endosomal/lysosomal membrane protein LAMP1 (Figure 2B). An augmented red autofluorescence of doxorubicin was also detected in the cytoplasm of the treated *GNPTAB* KO cells (by approximately 6-fold compared to the control). Furthermore, using the transfection of a LAMP1-GFP construct, we found that these red cytoplasmic signals were mainly located within lysosomes (Figure 2C). Therefore, it is possible that the confinement of weak bases in lysosomes contributes to the diminished effect of doxorubicin (and likely chloroquine too) in *GNPTAB* KO cells that cannot synthesize Man-6-P. Interestingly, staurosporine and paclitaxel, despite not being weak bases, also exhibited a reduced activity in *GNPTAB* KO cells, underlining the existence of alternative/additional resistance mechanism(s).

### 2.3. The IGF1R Pathway Is Hyperactivated in GNPTAB Knockout HeLa Cells

The anti-apoptotic effect of the IGF1R has long been recognized [24,25]. In addition to IGF-I, this receptor has a high affinity for IGF-II, whose bioavailability is controlled by endocytosis and lysososomal degradation. On the other hand, the receptor carrying out the IGF-II internalization is type 2 IGFR, or IGF2R, also known as the Cation-Independent Mannose-6-Phosphate Receptor (CI-M6PR). The latter mediates the endocytosis of IGF-II and secreted Man-6-P-bearing lysosomal acid hydrolases [26,27,28]. Moreover, acid hydrolases that carry multiple Man-6-P residues can bind to two neighboring IGF2R molecules, thus inducing their dimerization. This promotes IGF-II internalization, which, in turn, decreases its availability to the IGF1R [27,29]. In light of these findings, we postulated that the absence of Man-6-P signals on the acid hydrolases secreted by *GNPTAB* KO HeLa cells could reduce IGF2R dimerization and thus IGF-II internalization, thereby making this growth factor more available to the IGF1R, whose activation could, in turn, promote the resistance to apoptosis.

**Figure 3:**
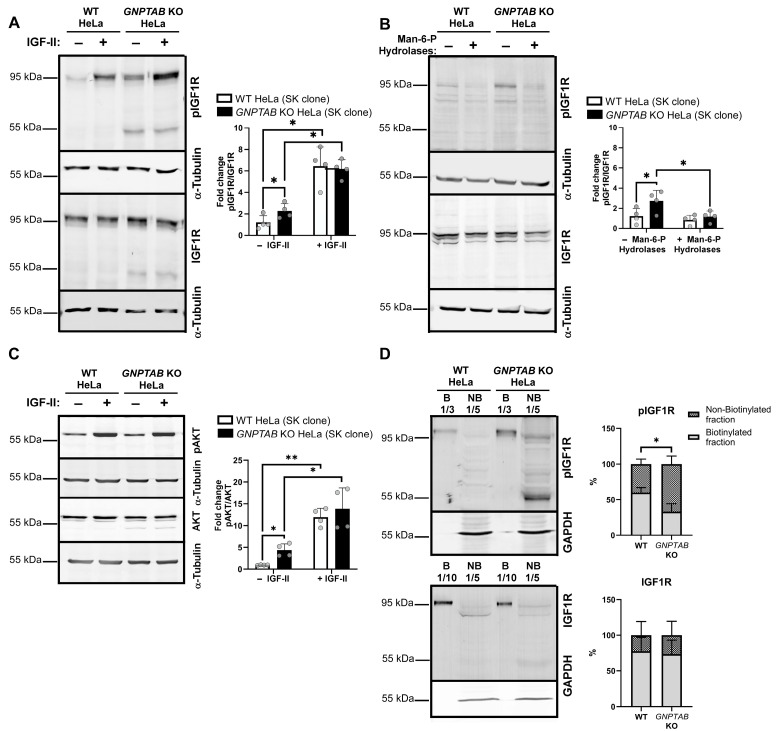
**IGF1R pathway activation in *GNPTAB* KO HeLa cells.** (**A**) Western blotting detection of total IGF1R and phosphorylated (Tyr1135/1136) IGF1R (pIGF1R) in lysates of WT and *GNPTAB* KO HeLa cells (SK clones) cultured in 10% FBS-containing medium supplemented or not with IGF-II (30 ng/mL) for 30 min. Graph shows mean relative level (fold changes) of pIGF1R/IGF1R ratio ± SD. n = 4 independent experiments. * *p* < 0.05. One-way ANOVA (**B**) Western blotting detection of total IGF1R and pIGF1R after 48 h of incubation of cells with purified Man-6-P-bearing acid hydrolases extracted from mouse brain. Graph shows mean relative level of pIGF1R/IGF1R ratio ± SD. n = 4 independent experiments. * *p* < 0.05. One-way ANOVA. The 55 kDa fragment detected in KO cells was not included in quantifications. (**C**) Western blotting detection of total AKT and phosphorylated (Ser473) AKT (pAKT) in conditions described in A. Mean relative level of pAKT/AKT ratio ± SD. n = 4 independent experiments. * *p* < 0.05. ** *p* < 0.01. One-way ANOVA. (**D**) Proteins located at cell surface were biotinylated (B fraction) at 4 °C and separated from non-biotinylated proteins (NB, intracellular fraction) as described in Section 4. GAPDH is cytosolic protein and was detected in these fractions to control that biotinylated reagent did not enter cells. As expected, no signal was detected in B fraction for this protein. pIGF1R and IGF1R were then analyzed. Dilution factors of each fraction are indicated at top of each blot. Graph shows percentage of signal detected in each fraction. n = 3 independent experiments. * *p* < 0.05. Unpaired *t*-test.

To test this hypothesis, the IGF1R activation was examined in the SK control and *GNPTAB* KO HeLa cells by detecting its activated form by Western blotting using an antibody that recognizes phosphorylated tyrosines on positions 1135/1136. These tyrosines are part of the tyrosine kinase domain, and their phosphorylation is necessary for receptor activity [30]. As shown in Figure 3A, we found a ~2-fold increase in the pIGF1R/total IGF1R ratio in *GNPTAB* KO HeLa cells compared to control cells under basal conditions. The treatment of both the control and KO cells with IGF-II was used as a positive control for the IGF1R activation by IGF-II. Importantly, we also frequently observed the presence of a ~55 kDa band in the KO cells, which likely represents an IGF1R degradation product accumulating in their lysosomes [31], with a diminished content of hydrolytic enzymes. In support of this interpretation, the treatment of the control HeLa cells with bafilomycin, which inhibits the vATPase-dependent acidification of lysosomes, yielded the same fragment (Supplementary Figure S3A).

The mouse brain is a rich source of Man-6-P-bearing acid hydrolases, due to the absence of acid phosphatase activity in the neurons [32]. A notable observation is that when the *GNPTAB* KO cells were incubated for 48 h with lysosomal enzymes purified from mouse brains using CI-M6PR affinity chromatography [32] (Figure 3B), their pIGF1R/IGF1R ratio became comparable to that of the control cells, implying that the activation of the IGF1R in KO cells was related to their deficient synthesis of Man-6-P. In further support, the increase in the pIGF1R level was reproduced in the *GNPTAB* KO clones newly generated by our group (Supplementary Figure S3B).

Next, we analyzed the levels of total and phosphorylated AKT, a downstream actor of the IGF1R pathway, both in the SK clones (Figure 3C) and in newly generated cells (Supplementary Figure S3C). An IGF-II treatment was used as a positive control for the activation of AKT via the IGF1R pathway. This analysis revealed that the pAKT/AKT ratio is also increased in *GNPTAB* KO cells compared to control cells.

Moreover, cell surface biotinylation assays conducted at 4 °C highlighted that, while the mature IGF1R is predominantly detected at the plasma membrane in both control and KO cells, the distribution of its phosphorylated form differs between these cell types. A total of 41 ± 5.3% of the pIGF1R was found in the non-biotinylated (i.e., intracellular) fraction of the control cells. This intracellular proportion increased to 62 ± 7% in *GNPTAB* KO cells (Figure 3D, *p* < 0.05). This increase was also detected in the newly generated clones (Supplementary Figure S3D).

Taken together, these data support the hyperactivation of the IGF1R pathway when the Man-6-P-dependent sorting to lysosomes is disrupted.

### 2.4. The IGF1R Signaling Pathway Is Involved in the Resistance of GNPTAB Knockout Cells to Apoptosis

**Figure 4:**
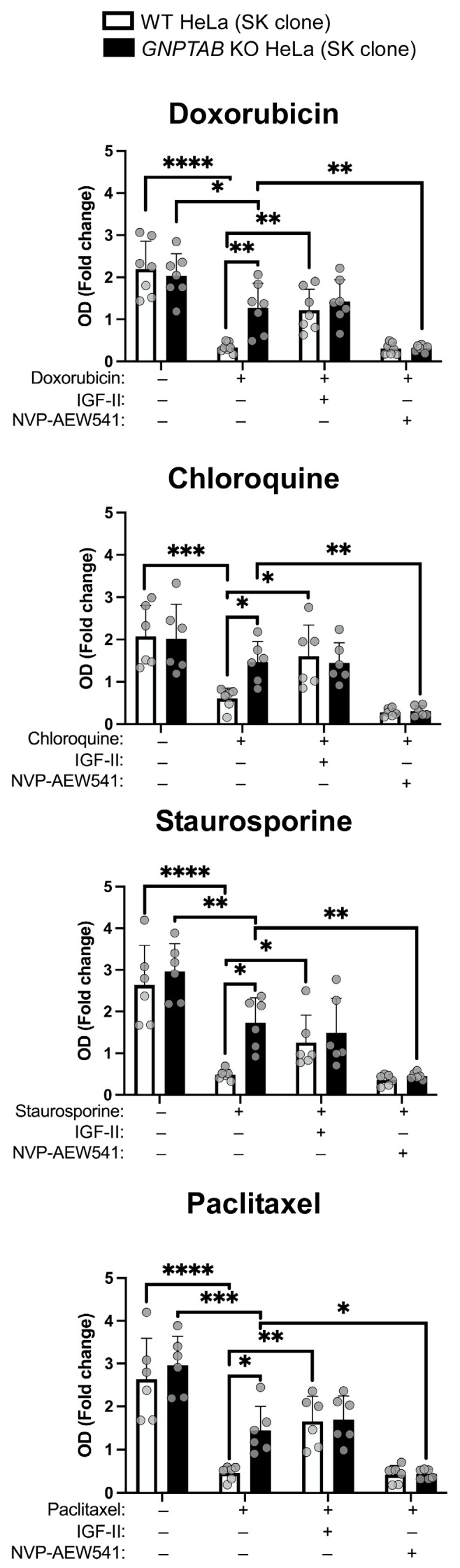
**Analysis of effect of IGF1R inhibitor on apoptosis resistance of *GNPTAB* KO HeLa**. Measurement of metabolic activity of WT and *GNPTAB* KO cells (SK clones) using MTT assay, 48 h after treatment with 2.5 μM doxorubicin, 25 μM chloroquine, 50 nM staurosporine or 50 nM paclitaxel in presence or absence of IGF-II (30 ng/mL) and of IGF1R inhibitor NVP-AEW541 (50 nM). n = 6 independent experiments. Means ± SD of OD_48h_ relative to OD_0h_ are shown on graph. * *p* < 0.05; ** *p* < 0.01; *** *p* < 0.001; and **** *p* < 0.0001. One-way ANOVA.

Lastly, we investigated whether treatment of the *GNPTAB* KO HeLa cells with NVP-AEW541, an IGF1R-specific tyrosine kinase inhibitor [33], could re-sensitize these cells to cytotoxic drugs. This inhibitor blocks the IGF1R autophosphorylation and the downstream signaling cascade and has been reported to disrupt IGF1R-dependent cell survival. Practically, we treated the cells with either doxorubicin, chloroquine, staurosporine or paclitaxel in the presence or absence of this inhibitor or in the presence or absence of IGF-II supplementation. The remaining metabolically active cells were then analyzed using an MTT assay. The data shown in Figure 4 reveal that SK *GNPTAB* KO cells, collected after 48 h of treatment with NVP-AEW541, lost their resistance to the four drugs. Moreover, the control HeLa cells exhibited an increased resistance upon treatment with IGF-II, most likely due their IGF1R activation. Similar a re-sensitization was observed for all four of the studied drugs (Supplementary Figure S4).

The results obtained for the 24 h and 72 h treatments are in Figure S4 (Supplementary). Figure S5 also displays the results of the control MTT experiment, showing that incubation with the inhibitor alone had no effect on cell proliferation, and the results of a Western blotting experiment demonstrating the inhibitory action of NVP-AEW541 on the IGF-II-induced phosphorylation of the IGF1R. We also validated the re-sensitization of the *GNPTAB* KO cells upon the treatment with the IGF1R inhibitor using the newly generated clones (Figure S5). Moreover, considering that the off-target effects of a chemical inhibitor can never be entirely excluded, we conducted an additional control experiment using another commercialized IGF1R inhibitor, NVP-ADW742 [34,35].

## 3. Discussion

Mucolipidosis type II (I-cell disease) and III are multisystemic disorders resulting from a deficiency in Man-6-P synthesis by GlcNAc-1-phosphotransferase and characterized by growth and developmental retardation, joint alterations, coarse features, valvular defects, osteoporosis, thickened skin and gingival hyperplasia [4,10,12,13,36]. Several of these manifestations are reproduced in mouse models with knocked-out and/or knocked-in *GNPTAB* or *GNPTG* genes, which code for GlcNAc-1-phosphotransferase subunits [37,38,39,40,41,42,43,44]. However, the disruption of the Man-6-P-dependent sorting of acid hydrolases by knocking-out the *IGF2R* gene, which codes for the CI-M6PR involved in the transport of Man-6-P-bearing acid hydrolases to the endo-lysosomal system, results in perinatal death in mice [45]. The mice can be rescued from death by knocking out the genes coding for IGF-II or one of its receptors, the IGF1R [46]. The other known IGF-II receptor is in fact the CI-M6PR/IGF2R. IGF-II binding to IGF2R mediates the IGF-II clearance from the extracellular space and its subsequent degradation in lysosomes. On the other hand, IGF-II binding to the IGF1R activates signaling cascades promoting cell proliferation, differentiation, migration and survival [24,25]. Thus, in knockout mice that do not express a functional CI-M6PR/IGF2R, increased circulating levels of IGF-II (due to its decreased clearance) lead to IGF1R hyperactivation and fetal developmental defects [46].

Moreover, IGF-II internalization by the CI-M6PR/IGF2R receptor is promoted by acid hydrolases that carry multiple Man-6-P signals. Indeed, Kornfeld et al. documented that β-glucuronidase, a lysosomal enzyme with many Man-6-P moieties, increased the rate of IGF-II internalization by cross-bridging two CI-M6PR/IGF2Rs [27]. IGF2R dimerization can also be triggered by synthetic compounds with multiple Man-6-P residues. Interestingly, increasing the clearance of extracellular IGF-II by the treatment with these Man-6-P multivalent molecules inhibited the proliferation of several cancer cell lines due to a decreased IGF1R activation [29].

Based on these findings, we assumed that the cell survival, promoted by the IGF1R, could be modulated by lysosomal acid hydrolases. Indeed, we have shown that the absence of Man-6-P residues on acid hydrolases in *GNPTAB* KO HeLa cells, which serve as a mucolipidosis type II cell model, confers resistance to doxorubicin, chloroquine, staurosporine and paclitaxel. In addition, we revealed that the level of activated IGF1R and its downstream target AKT were increased in the studied KO cells. Moreover, the treatment with an IGF1R inhibitor (NVP-AEW541 or NVP-ADW742) restored the sensitivity of these cells to all four tested drugs, despite these drugs having different chemical structures and acting through different mechanisms. Doxorubicin is a chemotherapeutic drug and DNA intercalating agent that inhibits topoisomerase II, thereby disrupting DNA replication and repair [47]. Chloroquine is well known as an anti-malarial agent but also exhibits anti-neoplastic properties in various cancers [48]. The mechanism of action of staurosporine is not entirely clear. This apoptosis-triggering molecule appears to be a potent and cell-permeable inhibitor of various protein kinases [49]. Lastly, paclitaxel is a generic name for a well-known chemotherapy drug called Taxol that prevents microtubule depolymerization. It activates the mitotic spindle assembly checkpoint, thereby inhibiting mitosis, and activates caspases [50].

Thus, the activation of the IGF1R pathway appears to be an important component of the apoptotic resistance of the Man-6-P sorting-deficient cells to apoptosis caused by the studied drugs. However, other factors contributing to this resistance cannot be excluded without additional study. One reason could be that the confinement of doxorubicin or chloroquine in lysosomes limits their cytotoxicity. However, it seems unlikely that this could significantly affect staurosporine- and paclitaxel-dependent apoptosis. Terman et al. [14] postulated that the disrupted Man-6-P synthesis and hypersecretion of cathepsins by fibroblasts isolated from mucolipidosis II patients, resulting in low levels of these enzymes in lysosomes, could prevent apoptosis initiation upon the treatment with cytotoxic drugs. Indeed, the apoptotic cascade can be enhanced by the release of cathepsins, including cathepsins B, D and L, upon the lysosomal membrane permeabilization [51,52]. However, intracellular levels of cathepsin D and L are higher in *GNPTAB* KO HeLa cells than in parental cells, although the cathepsin D maturation does not progress beyond its intermediate form, which is already active [53,54]. This is not unexpected as it has been documented that some acid hydrolases may be sorted to the lysosomes by Man-6-P-independent pathways [20,21]. In contrast, cathepsin B levels were found to be quite low in these KO HeLa cells [54]. Yet, Tardy et al. reported that the sensitivity of mucolipidosis II fibroblasts to cell death induced by staurosporine, sphingosine or TNF, acquired after these cells were exposed to a mixture of Man-6-P-bearing hydrolases, cannot be reversed by the incubation of the cells with a cathepsin B inhibitor. This finding suggests that a low cathepsin B activity is insufficient to confer resistance [55]. They also documented that cathepsin D-deficient fibroblasts are not protected from the action of cytotoxic agents [15]. An intriguing observation is that the supplementation of the medium with tripeptidyl peptidase 1 (TPP1), a lysosomal carboxypeptidase, could re-sensitize mucolipidosis II fibroblasts to the TNF-induced cell death [55]. It was later shown that this enzyme can cleave Bid, a central actor in the apoptotic cascade that leads to mitochondrial membrane permeabilization [56]. While we did not investigate whether TPP1 supplementation alone can re-sensitize *GNPTAB* KO HeLa cells to drug-induced cell death, the resistance of these cells to the different drugs completely diminished when they were incubated with the IGF1R inhibitor, suggesting that IGF1R is an initiating agent in this resistance pathway.

These new findings raise the possibility that the dysfunction of the IGF1R signaling cascade may contribute to the pathophysiology of mucolipidosis II and III, considering that the failure to remove and renew cells has severe consequences on tissue organization and functions [57,58]. Furthermore, an impaired acid hydrolase secretion has been reported in many major pathologies, including cancer, viral infections, neurological disorders, metabolic, musculoskeletal diseases and so on [59,60,61,62,63,64,65]. The underlying mechanisms may include the following, among other possibilities that remain to be explored: the altered expression of M6PR or its ligand binding efficiency; disrupted M6PR trafficking; overexpression of lysosomal enzymes, hence overloaded M6PRs; and augmented lysosomal exocytosis, [66,67,68]. Regarding the increased exocytosis of the lysosomal hydrolytic arsenal, enzymes lacking the Man-6-P moiety may also be released since the phosphate group in Man-6-P can be hydrolyzed by acid phosphatases originating from lysosomes of organs/tissues other than the brain [32,69]. Hence, our study offers novel insights into a potential mechanism contributing to the pathophysiology of diseases associated with altered lysosomal enzyme secretion. Specifically, it raises the possibility that alterations in the extracellular concentration of enzymes bearing or lacking Man-6-P may have differential consequences on the IGF1R pathway, either limiting or boosting anti-apoptotic cell protection, respectively, which could both have pathological consequences.

## 4. Materials and Methods

### 4.1. Cell Culture, Treatments and Transient Transfections

*GNPTAB* KO and parental HeLa cells were generously gifted by S. Kornfeld (Washington University in St. Louis, St. Louis, MI, USA). They were cultured in DMEM (Dulbecco’s Modified Eagle Medium, Lonza, Antwerp, Belgium) supplemented with 10% FBS (Fetal Bovine Serum, Merck, Hoeilaart, Belgium) and 100 units/mL of penicillin and 100 µg/mL of streptomycin (Lonza, Antwerp, Belgium). The cells were grown in a humidified atmosphere at 37 °C with 5% CO_2_. For drug resistance assessment upon time (24–48–72 h), cells were seeded in 15,000 cells/well in 96-well microplates (Greiner, Kremsmünster, Austria). Twenty-four h after seeding (time point considered as Day 0 of the experiment), cells were treated or not with doxorubicin (2.5 μM; ApexBio, Houston, TX, USA), chloroquine (25 μM; InvivoGen, San Diego, CA, USA), staurosporine (50 nM; Apollo Scientific, Stockport, UK) or paclitaxel (50 nM; Merck, Hoeilaart, Belgium). For detection of caspase 3/7 activity (as described below), treatments were applied for 8 h with 2.5 μM doxorubicin, for 24 h with 25 μM chloroquine or 50 nM paclitaxel or for 4 h with 50 nM staurosporine (these conditions allowed the earliest detection of caspase 3/7 activation). When indicated, culture media were supplemented with 30 ng/mL IGF-II (Merck, Hoeilaart, Belgium), 50 nM NVP-AEW541 (Merck, Hoeilaart, Belgium), 100 nM NVP-ADW742 (Merck, Hoeilaart, Belgium), 10 nM bafilomycin A1 (Merck, Hoeilaart, Belgium) and/or with purified acid hydrolases isolated from healthy mouse brains using CI-M6PR affinity chromatography [32]. Briefly, proteins were extracted from mouse brains using 1% triton X-100 in 50 mM imidazole, pH 6.5, and 0.15 mM NaCl then loaded on columns containing 1 mL of sepharose beads coated with CI-MPRs. Columns were washed with 0.1% triton X-100 in 50 mM imidazole, pH 6.5 and 0.15 mM NaCl, then with 6 mL of this buffer containing 5 mM glucose-6-P and finally with 6 mL of buffer containing 10 mM Man-6-P to elute the Man-6-P-bearing acid hydrolases retained on the beads. Transient transfections of LAMP1-mGFP plasmid (was a gift from Esteban Dell’Angelica (Addgene plasmid # 34831; http://n2t.net/addgene:34831; RRID:Addgene_34831)) [70] (accessed on 20 December 2024) were conducted using FuGENE**^®^** HD reagent (Promega, Leiden, The Netherlands) following the manufacturer’s instructions.

### 4.2. MTT Assay

Cell metabolic activity, as an indirect measurement of cell viability, was assessed using an MTT assay ([3-(4,5-dimethylthiazol-2-yl)-2,5-diphenyltetrazolium bromide], Roth, Karlsruhe, Germany). After treatment in 96-well plates (as indicated above), the cells were washed and incubated in 100 µL of PBS containing 0.5 mg/mL of MTT for 2 h. The resulting formazan crystals were then dissolved in 100 μL DMSO for 30 min. The optical density was measured at 570 nm using a microplate reader (Spectral Max I3; Molecular Devices, Sunnyvale, CA, USA). Data shown on graphs are normalized to Day 0 (i.e., the day at which the treatments were started, which is 24 h after cell seeding).

### 4.3. Caspase 3/7 Assay

To evaluate the activity of caspase 3/7 after treatments (as described above), cells were lysed in 10 mM TRIS-HCl, 0.1 M NaCl, 1 mM EDTA and 0.01% Triton X-100, pH 7.4, for 30 min on ice then incubated for 1 h at Room Temperature (RT) with the Z-DEVD-R110 substrate, a caspase 3/7 specific substrate, prepared at a final concentration of 50 µM in reaction buffer (20 mM PIPES, 4 mM EDTA, 0.2% CHAPS, pH 7.4). Fluorescence signals were then detected using a microplate reader (Spectral Max I3; Molecular Devices, Sunnivale, CA, USA) (495 nm excitation–525 nm emission). For normalization, protein concentrations were measured using the Bradford method (Bio-Rad, Lokeren, Belgium).

### 4.4. Engineering of Additional GNPTAB KO HeLa Clones

Knockout of the *GNPTAB* gene was conducted using a CRISPR-Cas9 method. Briefly, HeLa cells from ATCC (CCL-2) were transfected with three plasmids. The first one is the pX330-U6-Chimeric_BB-CBh-hSpCas9 and was a gift from Feng Zhang (Addgene plasmid # 42230; http://n2t.net/addgene:42230 RRID:Addgene_42230) [71] (accessed on 15 December 2024). This plasmid contains a cDNA sequence encoding the Cas9 protein and a cloning site in which we inserted the sequence coding for a guide RNA(5’-*GGTCAGAGAACAGATGAG*-3’) targeting exon 3 of *GNPTAB*. The second plasmid is the same one in which we inserted the sequence coding for a second guide RNA (5’-*GCAAACCGTCTTGGTTACAG*-3’) targeting intron 3 of *GNPTAB*. The third plasmid contains a puromycin-resistance gene (pTERT). The cell population transfected with these 3 plasmids was treated with puromycin for 1 week prior to cloning and screening by PCR using primers amplifying a fragment spanning exon 3 and intron 3 where the deletion is expected upon Cas9 action. Primer_F1 5’-*GGTAGTGAGAGAAAAGGGAAGAGTG*C-3’ and primer_R1 5’-*GGTTCCGTTGTGTTTTTCCCAAGG*-3’ were used. Clones showing no deletion or incomplete deletion were further screened by Sanger sequencing using primers that amplify the targeted region to test for possible frameshifts. Primer_F2 5’-*ACAACTCACAGAGGAGGTACTTG*-3’ and Primer_R2 5’-*GAGGGGCAAGTTTGTTTGGAT*-3’ were used. Importantly, control cells were subjected to the same transfection and antibiotic-mediated selection steps, except that no guide RNA was introduced in the system.

### 4.5. Western Blotting

Proteins extracted from whole cells lysates (in PBS supplemented with 1% of Triton and with protease and phosphatase inhibitors (cOmplete^TM^ and phosSTOP^TM^ tablets, Roche, Brussels, Belgium)) were resolved by SDS-PAGE in reducing conditions. Proteins were then transferred onto low fluorescence PolyVinylidene DiFluoride (PVDF, Merck, Hoeilaart, Belgium) membranes using a transfer apparatus (Bio-Rad, Lokeren, Belgium). Non-specific binding was blocked using 1% bovine serum albumin (BSA, Sigma-Aldrich, Hoeilaart, Belgium) diluted in TBS-T (0.1% Tween-20 in 10 mM TRIS-HCl, pH 7.4) for 1 h at RT. Membranes were then incubated with specific primary antibody solutions at 4 °C overnight under gentle and constant shaking. The following concentrations of primary antibodies were used: 1:1000 for the rabbit anti-IGF1R (D23H3, 9750, Cell Signaling, Leiden, The Netherlands); 1:1000 for the rabbit anti-phospho-IGF1R that recognizes phosphorylation on tyrosines 1135/1136 within IGF1R (3024, Cell Signaling, Leiden, The Netherlands); 1:1000 for the rabbit anti-AKT (9272, Cell Signaling, Leiden, The Netherlands); 1:1000 for the rabbit anti-phospho-AKT that recognizes phosphorylation on serine 473 within AKT (4060, Cell Signaling, Leiden, The Netherlands); and 1:10,000 for the mouse anti-α-tubulin (Merck, Hoeilaart, Belgium). After several TBS-T washes, the membranes were incubated with secondary antibodies (IRDye^R^ 680RD for Goat anti-Rabbit or IRDye^R^ 800CW for Goat anti-Mouse, LI-COR Biotechnology, Lincoln, NB, USA) at RT for 1 h. Signals were detected using the Amersham^TM^ Typhoon scanner (General Boston Electrics, Boston, MA, USA). Image processing and quantifications were conducted using the ImageJ software (version 2.0.0-rc-69/1.52p).

### 4.6. Fluorescence Microscopy Analyses

For endogenous LAMP1 detection, cells were grown on glass coverslips and then fixed for 10 min at RT using 4% paraformaldehyde diluted in PBS, pH 7.4. Cells were washed and permeabilized using 0.02% Saponin- 3% BSA in PBS for 10 min. Cells were then incubated for 1 h at RT with a rabbit anti-LAMP1 primary antibody (D2D11, Cell Signaling) diluted 100 times in 1% BSA in PBS. Next, cells were washed with PBS and incubated for 1 h at RT with an Alexa-Fluor488 anti-rabbit antibody (Thermofisher, Brussels, Belgium) and diluted 250 times in 1% BSA in PBS. For the analysis of doxorubicin presence in LAMP1–GFP-positive structures, the cells were only fixed with 4% paraformaldehyde, without permeabilization. Coverslips were then mounted using Mowiol^®^ reagent (Merck, Hoeilaart, Belgium). For the assessment of weak base accumulation in lysosomes, live cells were incubated for 15 min with 2.5 µM of acridine orange (Merck, Hoeilaart, Belgium).

Fluorescent signals were observed with a SP5 Leica confocal fluorescence microscope using a 40× objective with a 1.4 numerical aperture (Carl Zeiss Microscopy Deutschland, Oberkochen, Germany). Image processing and quantifications were conducted using the ImageJ software (version 2.0.0-rc-69/1.52p). To count LAMP1-positive structures and assess their diameter, we used the “Analyze Particle” function of this software. To quantify the acridine orange and doxorubicin signal intensity in the cytoplasm, we delineated the cell cytoplasm and then cleared the nuclear area before measuring the total red signal.

### 4.7. Cell-Surface Biotinylation Assay

The cells were kept on ice, washed with ice-cold PBS supplemented with 0.7 mM CaCl_2_ and 0.25 mM MgSO_4_ (PBS^++^, pH 8) and then incubated with 1 mg/mL of biotin NHS-SS-Biotin reagent (EZ-Link^TM^, sulfosuccinimidyl-2-[biotinamido]ethyl-1,3-dithiopropionate, Thermofisher, Brussels, Belgium) and prepared in PBS^++^, for 40 min on ice. The biotinylation was stopped by washing five times with ice-cold 50 mM glycine in PBS^++^. The cells were then lysed in 1% Triton X-100 in PBS supplemented with inhibitors of proteases and phosphatases (cOmplete^TM^ and phosSTOP^TM^ tablets, Roche, Brussels, Belgium). The biotinylated cell surface proteins were separated from non-biotinylated intracellular proteins using streptavidin agarose beads (Pierce^TM^, Thermofisher, Brussels, Belgium). Biotinylated proteins were eluted by 200 mM DTT in 1× Leammli’s sample buffer (10 min at RT then 5 min at 95 °C). Immunoblotting analysis was used to detect IGF1R and pIGF1R as described above. For IGF1R detection, 1/10th of the biotinylated fraction and 1/5th of the non-biotinylated fraction were loaded on the gel, while for pIGF1R detection, 1/3rd of the biotinylated fraction and 1/5th of the non-biotinylated fraction were analyzed.

### 4.8. Statistical Analyses

Results are presented as mean ± SD or SEM. For statistical analyses and graphs, we used GraphPad Prism software v.8.02. Unpaired t-tests were used when comparing two groups for a single measure. One-way Anova analyses were used when comparing the two groups of cells, treated or not with a drug.

## Data Availability

All data used in this article are included within the figures and Supplementary Materials files.

## Supplementary Materials

The following supporting information can be downloaded at https://www.mdpi.com/article/10.3390/ijms26083586/s1.
