# Supplementary material for: Disruption of Man-6-P-Dependent Sorting to Lysosomes Confers IGF1R-Mediated Apoptosis Resistance
Source: Int J Mol Sci. 2025 Apr 10;26(8):3586. doi: 10.3390/ijms26083586 (PMC12026698; doi:10.3390/ijms26083586)
Supplement: Supplementary file 1 [file ijms-26-03586-s001.zip › ijms-3562280-supplementary.pdf]

Fig. S1

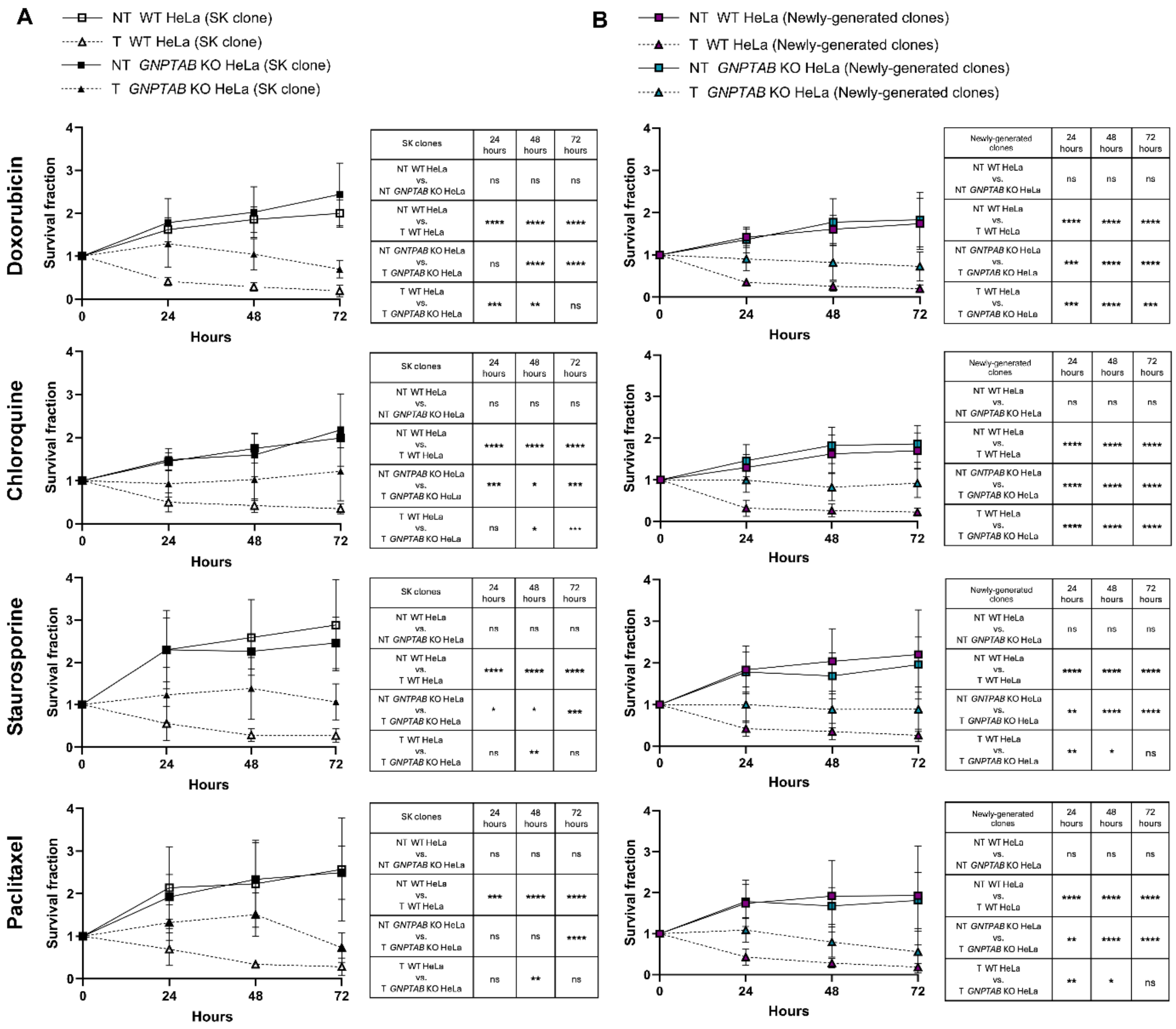

Supplementary Figure S1: **Time-dependent effect of doxorubicin, chloroquine, staurosporine and paclitaxel on control and *GNPTAB* KO HeLa clones.** After 24, 48 and 72 hours of treatment with 2.5 μM doxorubicin, 25 μM chloroquine, 50 nM staurosporine or 50 nM paclitaxel, a MTT assay was applied on control (WT, white) and *GNPTAB* KO (black) Hela cells provided by the S. Kornfeld's group (SK clones) (**A**), as well as on newly-generated clones including 2 control clones (pooled data; purple) and 3 *GNPTAB* KO clones (pooled data; green) (**B**). NT=Non-Treated (squares) and T=Treated (triangles) conditions. n=6 independent experiments for each individual clone. OD measured at a given time point relative to OD measured at day 0 (referred to as survival fraction) are shown on the graphs (means +/-SD). *p<0.05; **p<0.01; ***p<0.001; ****p<0.0001. One-way ANOVA.

Fig. S2

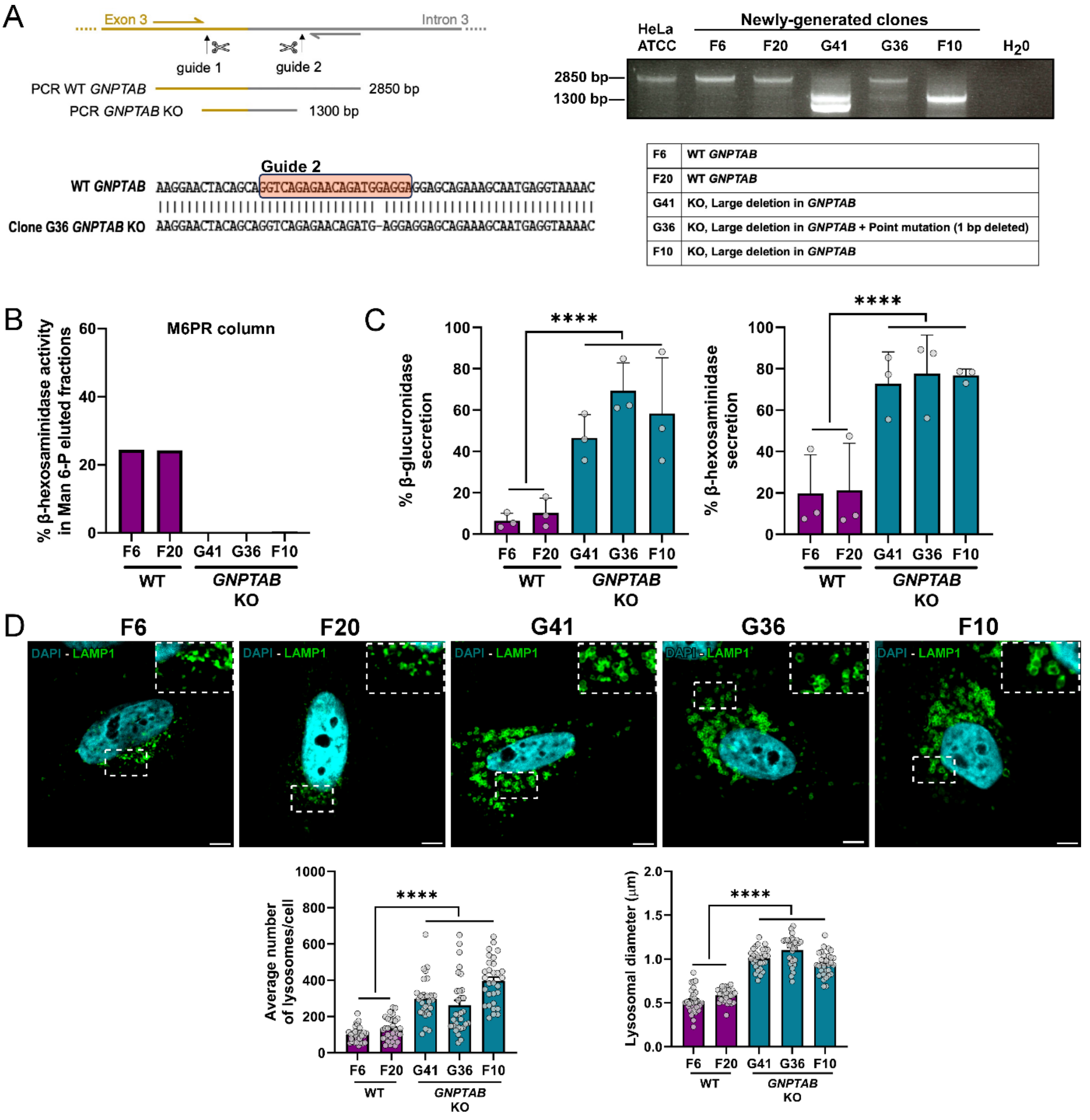

Supplementary Figure S2: **Characterization of newly-generated *GNPTAB* KO clones.** (**A**) Engineering of *GNPTAB* KO HeLa cells by CRISPR-Cas9 genome editing using guide RNAs (gRNAs) targeting exon 3 and intron 3, respectively. A deletion of ~ 1500 bp is expected if cleavage occurs at both sites, which is the case in clones G41 and F10 (see PCR screening in panel A). The G36 clone has an incomplete deletion status, but contains a frameshift in the remaining *GNPTAB* allele (see sequencing results in panel A). Two control clones (F6 and F20) were selected for comparison in subsequent tests. These cells have been subjected to the same manipulations, including antibiotic selection, but have unaltered *GNPTAB* sequence according to Sanger sequencing. (**B**) The presence/absence of Man-6-P signals on acid hydrolases secreted by control and *GNPTAB* KO clones (newly-generated) was assessed using a M6PR- affinity column. Non-specifically retained proteins on the column were removed using 5 mM of Glucose 6-Phosphate while proteins that bound specifically to M6PRs were eluted with 5 mM of Man-6-P. The graphs show the % of a selected lysosomal enzyme (β-hexosaminidase) specifically eluted with Man-6-P (relative to the total activity measured in the starting sample), i.e., the fraction of enzymes carrying Man-6-P signals. Note that this fraction is about zero in KO clones. (**C**) Measurement of the

specific activity of lysosomal ß-hexosaminidase and ß-glucuronidase in conditioned media of newly-generated control and KO clones (4 hours of culture without serum) using fluorogenic substrates: 4-methylumbellyferyl-N-acetyl-ß-D-glucosaminide and 4-methylumbellyferyl-ß-D-glucuronide hydrate substrates (respectively). Of note, secreted enzyme activities are expressed as % of total activity measured in cell lysates and culture media. The graph shows the data obtained for the two control clones (F6 and F20) and 3 KO clones (G41, G36 and F10), with n=3 independent measurements for each clone. Note the increased secretion levels for the KO cells. (**D**) Immunofluorescence detection of the late endosomal/lysosomal marker LAMP1 (green). Nuclei were stained with DAPI (blue). Scale bars, 5 μm. The graphs show the average number of lysosomes per cell +/-SEM as well as their average size +/-SEM, calculated from the analysis of 10 cells for each clone (2 control and 3 *GNPTAB* KO) per experiment, with n=3 independent experiments. The KO clones show increased number and diameter of lysosomes. ****p<0.0001. Unpaired t-test were applied in panels C and D.

Fig.S3

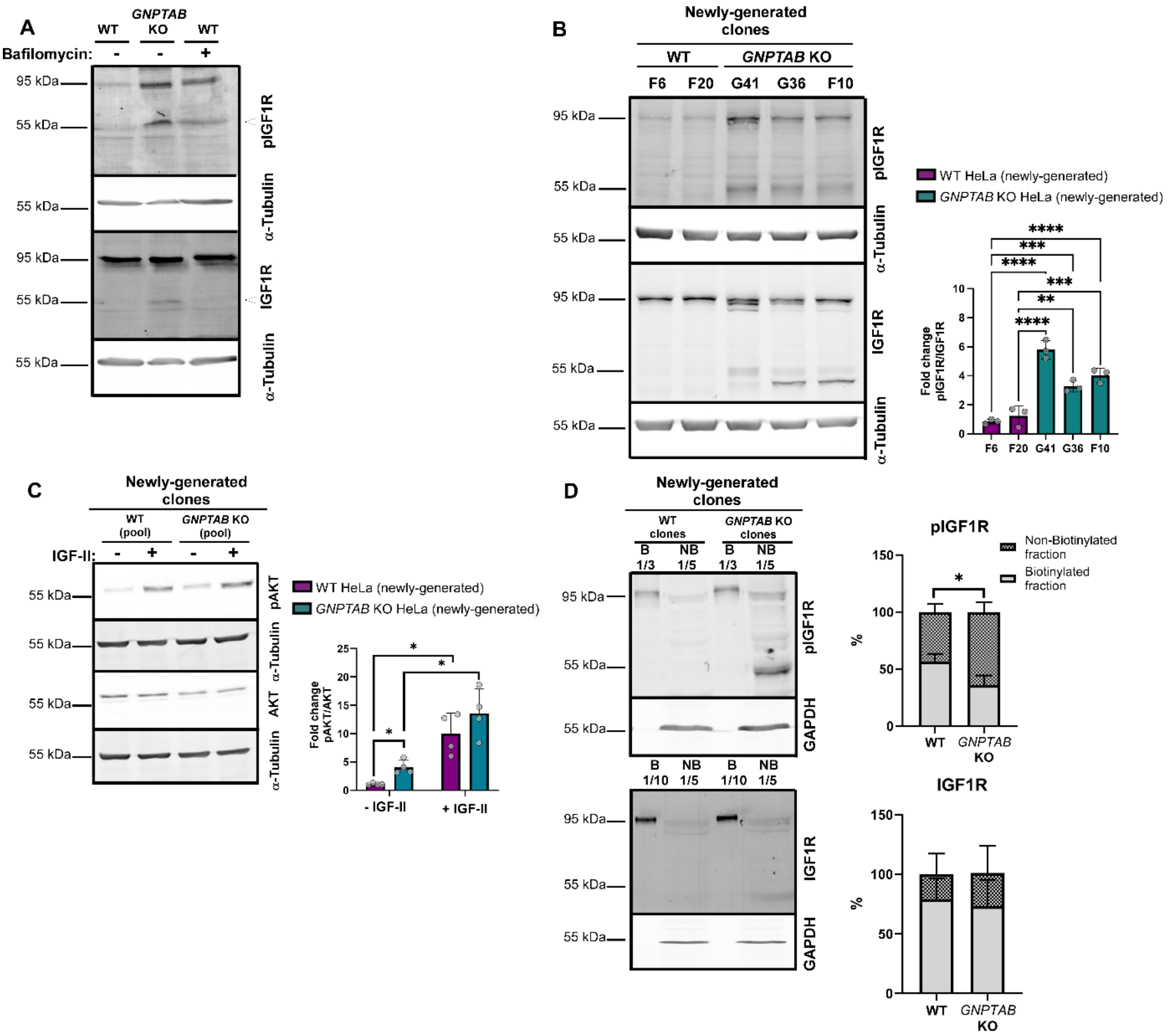

Supplementary Figure S3: **Controls associated with Figure 3.** (**A**) Bafilomycin treatment (10 nM for 16 hours) of control SK cells followed by the detection of IGF1R and pIGF1R by western blotting. Note the appearance of a fragment in treated cells, at the same molecular weight as the fragment observed in *GNPTAB* KO cells (~55 kDa, see arrowhead). (**B**) Western blotting detection of total IGF1R and phosphorylated (Tyr1135/1136) IGF1R (pIGF1R) in lysates of three newly-generated *GNPTAB* KO HeLa clones (G41, G36 and F10) under basal conditions, using two control clones (F6 and F20) for comparison. The graph shows relative level (fold change) of the pIGF1R/IGF1R ratio between control and KO groups. n=3 independent experiments. One-way ANOVA. (**C**) Western blotting detection of total AKT and phosphorylated (Ser473) AKT (pAKT) in lysates of the newly-generated cells treated or not with IGF-II (30 ng/ml) for 30 minutes. Of note, for this experiments, pooled cultures of the 2 control clones and of the 3 KO clones were used. The graph shows relative level (fold changes) of the pAKT/AKT ratio. n=4 independent experiments. One-way ANOVA (**D**) Proteins located at the cell surface of the newly-generated clones (pooled cultures) were biotinylated (B fraction) at 4°C and separated from non-biotinylated proteins (NB, intracellular fraction) as described in the Material and Methods section. GAPDH is a cytosolic protein and was detected by western blotting in these fractions to control that the biotinylated reagent did not enter the cells. As expected, no signal was detected in the B fraction for this protein. pIGF1R and IGF1R were then analyzed. The graph shows the percentage of signal detected in each fraction. n=3 independent experiments. *p<0.05. Unpaired t-test.

Fig.S4

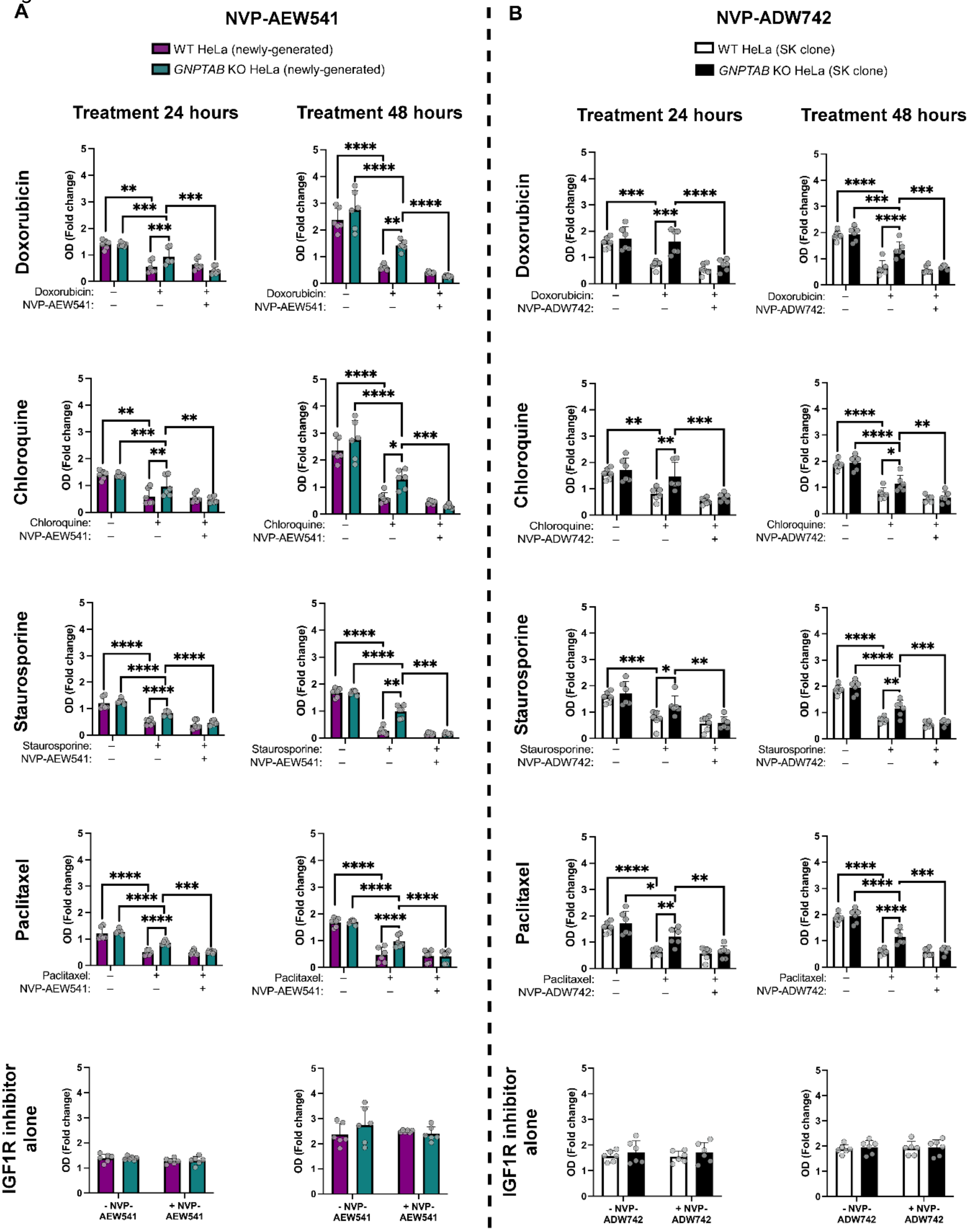

Supplementary Figure S5: **Analysis of the effect of IGF1R inhibition on *GNPTAB* KO HeLa cell resistance over time (additional controls).** Measurement of the metabolic activity of WT and *GNPTAB* KO cells using a MTT assay, 24 and 48 hours after treatment with 2.5 μM doxorubicin, 25 μM chloroquine, 50 nM staurosporine or 50 nM paclitaxel in the presence or absence of the IGF1R inhibitor NVP-AEW541 (50 nM) (panel **A**: newly-generated clones) or NVP-ADW742 (100 nM) (panel **B**: SK clones). Control of the absence of effect when incubated with each inhibitor alone is presented in panel A and B. n=6 independent experiments. Means +/-SD are shown on the graph. *p<0.05; **p<0.01; ***p<0.001; ****p<0.0001. One-way ANOVA.

Fig.S5

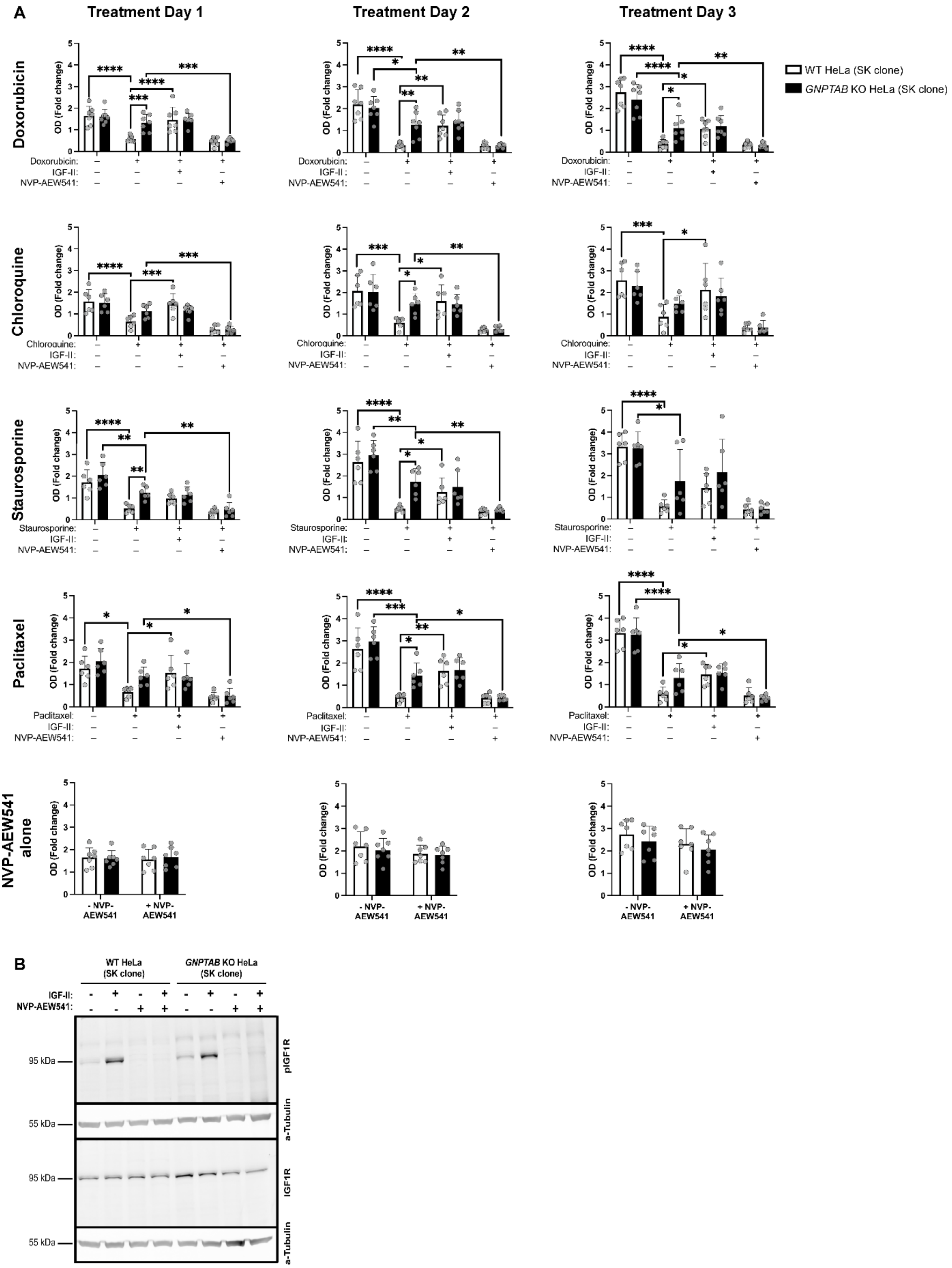

Supplementary Figure S4: **Analysis of the effect of IGF1R inhibition with NVP-AEW541 on *GNPTAB* KO HeLa cell (SK clone) resistance over time and control of NVP-AEW541 effect on IGF1R phosphorylation. (A**) MTT assay was conducted after 24, 48 and 72 hours of treatment with 2.5 µM

doxorubicin, 25 μM chloroquine, 50 nM staurosporine or 50 nM paclitaxel in the presence or absence of IGF-II (30 ng/ml) and of the IGF1R inhibitor NVP-AEW541 (50 nM). n=6 independent experiments. The graphs show controls of the absence of effect when incubated with inhibitor alone. (**B**) Western blotting detection of total IGF1R and phosphorylated (Tyr1135/1136) IGF1R (pIGF1R) in lysates of WT and *GNPTAB* KO HeLa cells cultured in the presence or absence of IGF-II (30 ng/ml) for 30 minutes and in the presence or absence of the IGF1R inhibitor NVP-AEW541 (50 nM) for 24 hours. n=3 independent experiments. Means +/-SD are shown on the graph. *p<0.05; **p<0.01; ***p<0.001; ****p<0.0001. One-way ANOVA. Statistical analysis were applied to analyze the difference.
